# Do Neurotrophins Connect Neurological Disorders and Heart Diseases?

**DOI:** 10.3390/biom11111730

**Published:** 2021-11-19

**Authors:** Masashi Fujitani, Yoshinori Otani, Hisao Miyajima

**Affiliations:** Department of Anatomy and Neuroscience, Faculty of Medicine, Shimane University, 89-1 Enya-cho, Izumo-shi 693-8501, Shimane, Japan; yotani@med.shimane-u.ac.jp (Y.O.); miyajima@med.shimane-u.ac.jp (H.M.)

**Keywords:** neurotrophins, nerve growth factor, brain-derived neurotrophic factor, tropomyosin-related kinase receptors, low-affinity p75 neurotrophin receptor, brain–heart axis, neurological diseases, cardiovascular diseases

## Abstract

Neurotrophins (NTs) are one of the most characterized neurotrophic factor family members and consist of four members in mammals. Growing evidence suggests that there is a complex inter- and bi-directional relationship between central nervous system (CNS) disorders and cardiac dysfunction, so-called “brain–heart axis”. Recent studies suggest that CNS disorders, including neurodegenerative diseases, stroke, and depression, affect cardiovascular function via various mechanisms, such as hypothalamic–pituitary–adrenal axis augmentation. Although this brain–heart axis has been well studied in humans and mice, the involvement of NT signaling in the axis has not been fully investigated. In the first half of this review, we emphasize the importance of NTs not only in the nervous system, but also in the cardiovascular system from the embryonic stage to the adult state. In the second half, we discuss the involvement of NTs in the pathogenesis of cardiovascular diseases, and then examine whether an alteration in NTs could serve as the mediator between neurological disorders and heart dysfunction. The further investigation we propose herein could contribute to finding direct evidence for the involvement of NTs in the axis and new treatment for cardiovascular diseases.

## 1. Introduction

Neurotrophins (NTs) are structurally homologous dimeric polypeptides and are one of the most characterized neurotrophic factor family members [1,2,3]. In mammals, NTs comprise nerve growth factor (NGF), brain-derived neurotrophic factor (BDNF), neurotrophin 3 (NT-3), and neurotrophin 4/5 (NT-4/5) [1,2,3]. NTs regulate the growth, development, survival, and regeneration of the nervous system. Their activity is mediated by two distinct types of receptors: tropomyosin-related kinase receptors (Trks) and low-affinity p75 neurotrophin receptors (p75NTRs) [1,2,3]. Trks display a high affinity and specificity for mature forms of NTs, whereas p75NTRs interact with all NTs, including pro-neurotrophins (pro-NTs). NGF preferentially binds to TrkA; BDNF and NT4/5 preferentially activate TrkB; and NT3 acts via all three receptors of the Trk family—TrkA, TrkB, and TrkC [1,2,3]. The affinity for pro-NTs is higher for p75NTR than for Trk [4].

Moreover, the prodomain of NTs was reported to be involved only in protein folding and secretion [5]. Subsequently, pro-NTs were shown to perform other functions and were found to be highly secreted by various tissues [6]. Pro-NTs are secreted from cells and processed by extracellular and intracellular cleavage to form mature NTs [7]. Importantly, pro-NTs perform biological functions mainly different from those of mature NTs [4]; several of these functions, such as inducing cell death and decreasing synaptic function, basically contrast with those of mature NTs [8]. However, under specific conditions, pro-NGF promotes the growth of neurites during postnatal development [9].

Although NTs are the most characterized trophic factors in the peripheral and central nervous systems, recently emerged, strong evidence suggests that NTs play important roles in cardiovascular systems [10,11,12,13]. In the early embryonic developmental stage, NTs, particularly BDNF, NT-3, and TrkB, and NT receptors are required in order to form a normal heart and vascular system [14,15,16,17]. In postnatal life, NTs control the survival of vascular smooth muscle cells (VSMCs) and endothelial cells (ECs) and regulate angiogenesis [12]. In addition to their function in controlling heart and vascular development, NTs regulate the innervation of sympathetic, parasympathetic, and sensory neurons during development [1,2,3]. NTs play an important role in regulating the autonomic and sensory nervous systems, acting not only as trophic survival factors, but also as regulators of cardiac nerve outgrowth and arborization [10,11,12,13], as well as musculoskeletal regulation [18].

**Figure 1:**
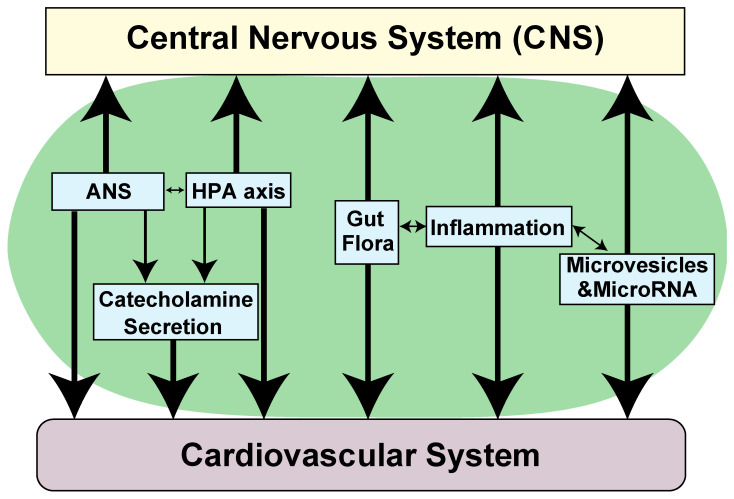
Summary of the brain–heart bidirectional interaction. Cardiac dysfunction and CNS disorders may be related by several mechanisms, including abnormal hypothalamic–pituitary–adrenal (HPA) axis, dysfunction in autonomic nervous system (ANS), catecholamine surge, gut dysbiosis, or systemic inflammation, as well as the release of microvesicles or microRNA.

**Figure 2:**
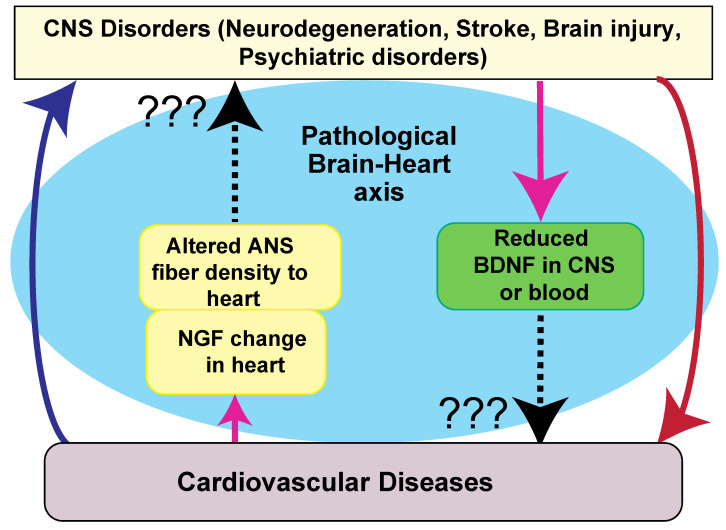
Summary of the potential mechanisms of the brain–heart axis mediated by NTs. As mentioned in Figure 1, cardiac dysfunction-related CNS disorders may be caused by several mechanisms. Epimediologically, CNS disorders are a risk factor of cardiovascular diseases (red downward arrow). Cardiovascular diseases can also be risk factors for CNS disorders (blue upward arrow). Regarding NT involvement, the NGF level and subsequent change in nerve density in the autonomic nervous system (ANS) are altered by cardiovascular diseases (pink upward arrow). Patients with neurological diseases show a reduced BDNF expression in the CNS and serum (pink downward arrow). However, it is still unkown whether these changes may induce both diseases (black dashed arrows + ???).

Our understanding of the effects of neurological disorders on the cardiovascular system or the influences of heart diseases on the CNS, the concept of so-called “brain–heart axis”, has recently expanded (Figure 1 and Figure 2) [19,20,21,22]. Recent evidence suggests that this axis signifies a bidirectional relationship between the cardiovascular function and neurological function. It is composed of potential mechanisms such as the hypothalamic–pituitary–adrenal (HPA) axis, autonomic nervous system (ANS), catecholamine secretion, gut flora, or systemic inflammation, as well as the release of microvesicles or microRNA (Figure 1).

On the one hand, how the heart influences the brain via NTs has been relatively well-discussed [13,23,24]. On the other hand, although growing evidence suggests a complex interactive association between CNS disorders and cardiac dysfunction [19,20,21,22], it is still unclear whether NTs are involved in these mechanisms.

To try to address this issue, in the first half of this review, we discuss the physiological function of NTs not only in the nervous system, but also in the cardiovascular system. In the second half, we discuss the involvement of NTs in the pathogenesis of adult cardiovascular diseases. Finally, we examine whether NT signaling plays a role in the brain–heart axis, with proposals for future research and treatment.

## 2. NTs Regulate Cardiovascular Function, Development, and Angiogenesis

### 2.1. The Physiological Role of NTs in the Cardiovascular System or Autonomic Nervous System

Okada et al. demonstrated that systemic deletion of BDNF or disruption of TrkB in the adult heart worsens cardiac dysfunction after myocardial infarction (MI), as described in the following sections. They concluded that brain-derived BDNF has a protective effect on myocardial remodeling after MI through a CNS-mediated mechanism [25]. This was the first evidence that the brain–heart axis is modulated by NTs. Another study showed that the activation of TrkB with agonistic antibodies results in a dose-dependent decrease in body weight and a transient increase in systolic and diastolic blood pressure in mice with diet-induced obesity [26].

Feng et al. analyzed cardiac-specific TrkB-knockout mice. In these mice, cardiac movement was impaired, indicating that BDNF/TrkB signaling regulates the maintenance of myocardial function itself, as well as heart development [27]. Additionally, Fulgenzi et al. showed that BDNF regulates cardiac contraction force independently of the innervation [28]. They used TrkB.T1 isoform-specific knockout mice. The TrkB.T1 isoform lacks the tyrosine kinase domain, but when BDNF binds to this receptor, it transmits signals, including those of phospholipase C pathways. These mice develop cardiomyopathy in adulthood, which is consistent with the observation of TrkB.T1 isoform expression in adult cardiomyocytes [28]. TrkB.T1 is activated by BDNF produced by cardiomyocytes, suggesting the existence of an autocrine/paracrine loop [28].

Recent findings indicate that BDNF also plays a major role in the autonomic nervous system control of cardiovascular function in adults [29]. Exercise and dietary energy restriction increase BDNF levels in the CNS and decrease heart rate and increase heart rate variability through augmenting the parasympathetic activity in the heart [29]. BDNF regulation of cardiovascular function also occurs via signaling in the central autonomic nuclei of the brainstem, hypothalamus, amygdala, or nucleus tractus solitarius (NTS) [29]. BDNF is expressed in both baroreceptors and chemoafferents in the nodose and petrosal sensory ganglia, which terminate in the brainstem [30,31]. Injecting BDNF into the NTS of anesthetized rats increases blood pressure, heart rate, and lumbar sympathetic nerve activity [32]. Conversely, inhibition of tonic BDNF signaling in the NTS with a TrkB receptor antagonist decreases blood pressure and heart rate, indicating that BDNF signaling in the NTS tonically modulates cardiovascular regulation [32].

### 2.2. NTs Regulate Heart Morphogenesis Directly and Indirectly

Based on the RNA-seq results provided by the Deciphering the Mechanisms of Developmental Disorders (DMDD) Program (http://www.dmdd.org.uk/, accessed on 6 October 2021), we summarize the gene expression patterns of mouse NTs and their receptors, as shown in Table 1. NT genes are expressed in the cardiovascular system as early as embryonic day 10. These results are consistent with those of earlier studies on the gene expression of NTs and their receptors in rats [33,34], as well as studies using knockout or transgenic mice, as described below.

In NT3 knockout mice, abnormalities were first observed in the greater vessels at E9.5, with reduced circumferential myofibrillar orientation in the truncus and abnormal cardiac morphogenesis [16]. Subsequently, impaired cardiac morphogenesis, including globular heart, atrial septal defect (ASD), ventricular septal defects (VSD), pulmonary stenosis, truncus arteriosus, and valvular defects, was observed in TrkC knockout mice at postnatal day (P) 0 [35]. In addition to NT3/TrkC signaling, BDNF/TrkB signaling is involved in cardiac development.

BDNF knockout mice showed intracardiac hemorrhage and reduced cardiac contraction ability at P0 [14]. Conversely, in mice overexpressing BDNF, the capillary density was increased [14]. TrkB knockout mice also showed a marked decrease in blood vessel density and increased apoptosis of endothelial cells (ECs) in the subepicardial region of the E12.5 heart [15]. Conditional deletion of TrkB in SMCs resulted in a marked reduction in pericyte/SMC density in the heart and caused perinatal lethality [36]. These findings suggest that BDNF/TrkB signaling plays an important role in the regulation of pericytes/SMCs during development [36].

From E11.5 to 12.5, neural crest-derived cells begin to migrate, and play an important role in the development of cardiac innervation [37]. This suggests that NTs have direct effects on cardiovascular cells, because some of these developmental defects appeared earlier than the development of cardiac innervation from E11.5 [12,37].

In addition to in vivo studies, in vitro studies showed that Trk receptors were present on vascular ECs [38], vascular SMCs [39], and cardiomyocytes [40,41]. p75NTR is expressed in ECs [38,42] and SMCs [43]. In normal cardiomyocytes, the p75NTR and pro-NGF expression was found to be very low [44].

NTs affect cardiovascular development via the nervous system [45], as cardiac muscle-specific NGF overexpression induces cardiac hypertrophy via hyperinnervation [45]. In contrast, NGF and TrkA knockout mice show few morphological abnormalities in cardiovascular development [12,46]. Therefore, the NGF/TrkA signaling pathway is important for sympathetic nerve growth, survival, differentiation, patterning, and synaptic strength rather than direct regulation of cardiovascular morphogenesis [11,45].

### 2.3. NTs Regulate Angiogenesis

Angiogenesis plays a pivotal role not only in physiological development from embryo to adult, but also in pathological conditions, as described below. To study angiogenesis during development, researchers developed a method using the chick embryo chorioallantoic membrane (CAM), iris, and blood vessel-free cornea in rodent eyes [47]. NGF was found to induce a dose-dependent angiogenic response in the rat cornea [48] and chick and quail embryo CAM [38,49]. Interestingly, NGF-induced neovascularization was inhibited by neutralizing anti-NGF antibodies, but was only partially affected by anti-vascular endothelial growth factor (VEGF) antibodies [38,49]. These findings support the idea that there may be a crosstalk between NGF and VEGF signaling. Emanueli et al. showed that NGF promotes angiogenesis in response to an ischemic limb [50].

Raychaudhuri et al. first described the proliferative effects of NGF in human dermal microvascular ECs [51]. Subsequently, TrkA-mediated survival of ECs was confirmed in human umbilical vein ECs [38], human choroidal ECs [52], and rat brain ECs [53]. Moreover, NGF-mediated migration of ECs has been revealed by several studies [54,55,56]. The migration of ECs by NGF was not mediated by VEGF signaling [56]. With respect to signal crosstalk with VEGF, Hansen-Algenstaedt et al. revealed more evidence that the overexpression of NGF increased both VEGF and VEGF-Rs in brown adipose tissue [57].

In non-ischemic conditions, BDNF induced the recruitment of cells expressing markers of ECs, VSMCs, and macrophages in Matrigel, and a sustained increase in vascular density was observed in response to BDNF in a mouse non-ischemic ear model [58]. In an ischemic hindlimb model, delivery of BDNF with adenoviral vector enhanced revascularization [58]. This effect required the expression of TrkB and involved the specific recruitment of ECs, monocytes/macrophages, and bone marrow-derived pro-angiogenic hematopoietic cells [58].

Subsequent studies have shown that the expression of TrkB isoforms in cardiac microvascular cells is dynamic, with cells from young animals expressing kinase-active TrkB and those from aged animals predominantly expressing the truncated TrkB.T1, which lacks the tyrosine kinase domain. This suggests that the angiogenic capacity of BDNF is reduced in the hearts of aged animals [59].

A study by Cristofaro et al. suggested that NT-3 regulates EC proliferation, survival, migration, and network formation in vitro, and is involved in angiogenesis in a limb ischemia model [60].

## 3. Pathophysiological Role of NTs in Primary Cardiovascular Diseases

Consistent with the important role of BDNF in physiological cardiac function as described above, the Framingham Heart Study (FHS) showed that serum BDNF was inversely associated with cardiovascular risk and mortality [61]. SNP rs6265 was found to be associated with BDNF concentrations and coronary artery disease in the FHS [61]. However, the functions of NTs in pathological cardiovascular diseases need to be discussed.

### 3.1. Ischemic Injury

NGF is expressed in the normal heart, as shown in Table 1 and [34], but its synthesis and release are altered under various pathological conditions [10,11,12,13]. NGF is an important factor in myocardial response to ischemia. Under these conditions, the expression of NGF and TrkA is upregulated in the human heart [62] and in rat experimental models [63]. As mentioned above, NGF promotes sympathetic hyperinnervation, angiogenesis, and cell survival. In mice, NGF gene transfer into the infarcted myocardium increases the survival of both ECs and cardiomyocytes, stimulates neovascularization, and improves myocardial blood flow [62]. Cardiomyocytes, myofibroblasts, and macrophages have also been identified as sources of NGF [64,65].

BDNF, but not NT-3, was shown to be rapidly induced after transient myocardial ischemia [63]. BDNF levels, but not NT-3, in the unstable angina group had significantly greater differences between the coronary sinus and aorta than those in the stable effort angina or non-coronary artery disease groups [66]. Consistent with its serum levels, the BDNF expression in atherosclerotic coronary arteries was upregulated [66]. More recent studies have shown that BDNF and pro-BDNF proteins are induced in mouse hearts after left coronary artery occlusion, and that plasma BDNF is increased in patients after acute MI [67].

As mentioned above, in an ischemic hindlimb model, the administration of an adenovirus harboring BDNF enhanced revascularization [58]. To directly assess the potential therapeutic effects of BDNF in the ischemic heart, several studies have used intramyocardial injections of the BDNF protein. Contradictory results have been obtained [59,67,68]; therefore, further investigation is necessary to determine the therapeutic effects of BDNF after MI.

A study using BDNF haploinsufficient mice reported improved survival of mice and reduced negative remodeling of the infarcted area [69]. Additionally, in BDNF haploinsufficient mice, the initial influx of neutrophils was reduced, the subsequent influx of macrophages into the infarct regions was enhanced, and neovascularization was reduced [69].

In addition, Okada et al. demonstrated that the conditional deletion of BDNF decreased the ejection fraction and increased the fibrosis area after MI, suggesting that BDNF plays a cardioprotective role [25]. The authors also examined mice with a conditional deletion of TrkB in adult cardiomyocytes. They found a statistically significant decrease in the ejection fraction and an increase in cardiac fibrosis after MI [25]. They also confirmed that conditional deletion of BDNF from cardiomyocytes in adult mice did not affect cardiac function post-MI in these mice [25].

Taken together, these studies suggest that BDNF plays an important role in orchestrating the repair mechanisms after MI, with distinct actions, such as hematopoietic cell recruitment, EC cell survival, maintenance of cardiomyocyte function, and cardiac innervation.

In contrast, Siao et al. described the induction of pro-NGF by cardiomyocytes and p75NTR in human arterioles after MI [44]. In myocardial ischemia-reperfusion injury in mice, rapid upregulation of pro-NGF by cardiomyocytes and p75NTR by microvascular pericytes has been observed [44]. A mouse expressing pro-NGF showed activation of the cardiac microvascular endothelium, decreased pericyte process length, increased vascular permeability, and lethal cardiomyopathy in adults [44]. These studies suggest that pro-NGF is involved in ischemic injury.

### 3.2. Heart Failure (HF)

In contrast with the increased cardiac NGF observed immediately after MI, as myocardial damage progresses to congestive heart failure, NGF production is reduced in humans, rats, and dogs [70,71,72]. In a zebrafish model of cardiac injury, NGF reduced the incidence of heart failure and mortality [73]. In a mouse model of heart failure associated with diabetic cardiomyopathy, NGF gene therapy with adeno-associated vectors has been shown to be a potential drug candidate [74]. The diabetes-induced decline in cardiac function is prevented by NGF overexpression [74]. Moreover, increased cardiac NGF expression prevented the enlargement of the left ventricular (LV) chamber volume and maintained the LV internal diameter [74]. These data suggest that NGF can be a relevant factor for promoting cardiac regeneration and angiogenesis in the failing heart.

There is mounting evidence that sympathetic efferent neuronal activity is increased in chronic HF [23]. In severe heart failure, continuous norepinephrine infusion induces a loss of sympathetic fibers, presumably because of NGF reduction [71]. Taken together, although NTs are involved in the pathophysiology of heart failure, it remains unclear whether CNS disorders are caused by autonomic nervous system hyperactivation as described below [13,23,24] (Figure 2).

A recent case-control study by several groups demonstrated that serum BDNF levels were lower in patients with heart failure and reduced ejection fraction [75,76]. Importantly, it is still unclear whether CNS is involved in BDNF reduction, because the non-neural expression of NTs has been frequently observed as described in several review articles [77,78].

### 3.3. Doxorubicin-Induces Cardiotoxicity

Doxorubicin (DOX)/adriamycin is effective in the treatment of several cancers; however, dose-dependent cardiac adverse reactions, such as heart arrhythmias, irreversible cardiomyopathy, and congestive heart failure, significantly limit its clinical use [79]. To overcome this, several groups have shown that NTs are involved in the pathogenesis of cardiotoxicity, and NTs play a protective role against cardiac damage through survival signaling [80,81].

## 4. Pathophysiological Role of NTs in Cardiovascular Abnormalities Associated with Neurological Diseases

As mentioned above, our understanding of the effects of neurological disorders on the cardiovascular system or heart diseases on neurological function has recently expanded (Figure 1 and Figure 2) [19,20,21,22].

First, regarding the effects of heart diseases on neurological function, epidemiological evidence suggests that heart disease may be a risk factor of Alzheimer’s disease (AD) [82,83], and it has been well accepted that heart diseases are a risk factor of stroke [84]. A higher prevalence of mental diseases in coronary heart disease patients has been demonstrated [85].

As discussed, regarding the mechanism, NGF derived from cardiomyocytes can control sympathetic nerve density when the heart is damaged by heart failure or cardiac hypertrophy [13,23,24] (Figure 2). Therefore, it has been relatively acceptable that the influences of heart diseases on the CNS are mediated by the autonomic nervous system with NTs (Figure 2). However, it is still elusive whether this NT-mediated autonomic modulation could cause neurological disorders.

Contrarily, although growing evidence suggests the effects of CNS disorders on cardiac dysfunction [19,20,21,22], it is still unclear whether dysregulation of NTs directly causes cardiac diseases (Figure 2).

### 4.1. Pathophysiological Role of NTs in Neurological Diseases and How to Modulate BDNF Expression

To start the discussion, we first narrowed the types of neurological diseases only to diseases affecting NT signaling in the brain. The changes in NGF [86,87] and BDNF [88,89,90] have been well discussed in neurodegenerative diseases, stroke, brain injury, and neuropsychiatric disorders. NT-3 or NT4/5 are less discussed in the pathogenesis of brain disorders than NGF or BDNF. Therefore, in this section, we will discuss cardiovascular dysfunction related to NGF- or BDNF-compromised brain diseases, such as AD or Parkinson’s disease (PD).

Interestingly, in animal models, it has already been established that BDNF can be the potential therapeutic target for psychiatric disorders and neurological disorders, even though the BDNF protein itself has a short half-life and can hardly penetrate the blood brain barrier [91,92,93]. How to modulate BDNF expression are well-described in excellent reviews [93,94], therefore, methods such as TrkB agonists, transplantation of BDNF-expressing cells, gene therapy of BDNF-expressing viral vectors, and BDNF-increasing factors such as exercise may be applied to treat heart disorders and neurological disorders.

### 4.2. Pathophysiological Role of NTs in Cardiovascular Abnormalities Associated with Neurodegenerative Diseases

Cardiac changes are commonly seen in neurodegenerative diseases, particularly synucleinopathies, such as PD, dementia with Lewy bodies, and multiple system atrophy [95,96]. A large network of cortical and brainstem regions controls cardiovascular function via the sympathetic and parasympathetic nervous system [10,11,12,13]. However, each pathological mechanism affecting cardiac function due to neurological diseases seems to differ in each disease [19,20,21,22].

In clinical studies, patients with PD showed reduced BDNF mRNA in substantia nigra [97,98]. The serum BDNF is lower in PD patients, and the severity of motor disability correlates with a lower serum BDNF [99,100]. A more recent study also revealed cardiovascular dysfunction related to reduced serum BDNF levels [101].

PD animal models exhibited cardiovascular dysfunction, such as reduced ^125^I-MIBG (metaiodobenzylguanidine) uptake, abnormal norepinephrine turnover, and depressed contractility, similar to human patients [102,103].

Therefore, elevated levels of BDNF in the serum or brain significantly improved motor functions and could rescue the cardiac dysfunction in PD animal models or patients [89,104]. If cardiac function is improved by increasing the BDNF level in the CNS of the PD model animal, it would provide strong evidence supporting the mediation of cardiac function by BDNF via the brain–heart axis.

Regarding AD, abnormal myocardial function with intramyocardial deposits of Aβ was observed in patients with AD [105,106,107]. In addition, a decreased protein level of BDNF was observed in the brains of AD patients, and the serum BDNF level was also found to be decreased [108,109]. Interestingly, the AD animal model, APPswe/PS1dE9 mouse (double transgenic mice overexpressing FAD-linked amyloid precursor protein with Swedish mutation and PS-1 with deletion of exon 9), did not show a consistent decline in brain BDNF levels as with human AD brains [109]. However, this animal model showed cardiomyocyte contractile dysfunction and reduced responsiveness to adrenergic agonists [110]. As mentioned above, increasing the BDNF level in the brain may rescue the cardiac dysfunction in AD model animals as the memory function was improved [111,112].

On the other hand, Shityakov et al. hypothesized and explained the link among age-related hearing loss (ARHL), AD, and the development of AD-related amyloid cardiomyopathy [22]. This is because AD, ARHL, and cardiac dysfunction are all affected by neurotrophic factor dysregulation, which may be due to the age-related imbalance of neurotrophic factors [22,89]. Therefore, rescue experiments by increasing BDNF level in the CNS may also reveal valuable insights into the relationship.

There is growing clinical evidence that cardiac dysfunction is linked to Huntington’s disease (HD) [113,114]. Critchley et al. revealed that HD model mice started to show obvious molecular and physiological heart abnormalities, such as BDNF mRNA reduction, in the nervous system [113]. This evidence might not support the idea that neurological diseases directly affect heart function, but it supports the idea that neurodegenerative diseases are systemic disorders, and neurotrophic factor dysregulation may be involved in their pathogenesis.

### 4.3. Pathophysiological Role of NTs in Cardiovascular Abnormalities Associated with Stroke and Brain Injury

Cardiovascular complications, including ischemic stroke, brain hemorrhage, and subarachnoid hemorrhage, are the second leading cause of post-stroke death [20]. Stroke-induced cardiac damage may lead to varying degrees of severe cardiac dysfunction, such as neurogenic stress cardiomyopathy, and Takotsubo cardiomyopathy (TTC). TTC is a transient LV apical ballooning syndrome that is triggered by stroke. It is diagnosed by elevated cardiac enzymes, wall motion abnormalities, and ECG changes [20].

The underlying mechanisms of brain–heart interactions after stroke have been well discussed [20,115,116]. The mechanism consists of abnormal upregulation of the hypothalamic–pituitary–adrenal axis, autonomic nervous system upregulation, and catecholamine surge, as well as gut microbiome and systemic immunological reactions against inflammation [19,20]. Biomarkers of cardiac damage and dysfunction after stroke have also been investigated, and the amino-terminal fragment of probrain natriuretic peptide (NT-proBNP) might be one of the best markers for cardiac damage after stroke [117]. Interestingly, epidemiological studies have shown that circulating concentrations of the BDNF protein are lowered in the acute phase of ischemic stroke, and low levels are associated with a poor functional outcome and mortality [118,119]. In experimental animal models, the NTs expression was increased after ischemic stroke [120].

NTs have not been shown to be involved in the underlying process of cardiac dysfunction after stroke. However, there is evidence that NTs play a role in the hypothalamic–pituitary–adrenal axis [121,122] and systemic immunological reactions against inflammation [123]. Therefore, further studies to ameliorate cardiovascular dysfunction by increasing the expression of BDNF in the CNS of animal models or human may support the concept of brain–heart axis [93].

The relationship between brain injury and heart dysfunction is relatively less strong than that between stroke and heart dysfunction [124,125]. However, the shared symptoms between traumatic brain injury and stroke, such as TTC, are well accepted in both diseases [126]. The mechanisms, such as hypothalamopituitary dysfunction, are also shared [127].

### 4.4. Pathophysiological Role of NTs in Cardiovascular Abnormalities Associated with Psychiatric Disorders

Epidemiological evidence and the underlying mechanisms of brain–heart interactions in depression have been well discussed [128,129]. As discussed in stroke and brain injury, inflammation, elevated catecholamine levels, abnormalities in the HPA axis, autonomic nervous system abnormality, and dysregulation of NTs have been proposed [128].

Many studies have revealed that BDNF is involved in chronic stress and psychiatric disorders, such as bipolar disorder, mood disorders, and schizophrenia [88,89,130,131]. NGF has also been shown to be involved in the pathogenesis of anxiety-like behaviors, chronic stress, and depression [87,131,132]. Recent advances have shown that NTs are involved in the neuroimmune system and play a crucial role in the regulation of psychiatric disorders [88,133]. In animal models, infusion of BDNF could reduce depression-like behavior, and increased expression of BDNF counteracts the effects of stress [134]. Although the effect of BDNF in each psychiatric regulation is highly network-dependent [134,135], the ectopic expression of BDNF might be applicable for the treatment of cardiac disorders with psychiatric diseases.

Through epidemiological studies, there is substantial supporting evidence that psychiatric disorders are associated with cardiac diseases via BDNF [85,136,137]. However, direct psychosomatic connections via NTs have not yet been fully revealed [128,132]. As mentioned above, the ectopic expression of BDNF in animal models could prove this idea.

## 5. Conclusions

Taking all of the above discussions into account, neurological disease patients seem to more easily develop cardiovascular dysfunction, and heart disease patients seem to develop more neurological diseases. Therefore, there should be an inter- and bi-directional relationship between CNS disorders and cardiac diseases via various potential mechanisms, including changes in NTs protein expression. To support the idea, the alteration of NTs is frequently observed in both diseases. However, further investigation is needed to conclude that NTs are a direct mediator between these two disorders.

Are there possibilities that other neurotrophic factors, such as the glial-derived neurotrophic factor family, acidic fibroblast neurotrophic factor family, and insulin-like growth factor, are involved in the brain–heart axis? These neurotrophic factors regulate heart development and/or function [11,138,139]. Therefore, the brain–heart axis may involve these molecules.

However, to our knowledge, investigation of the involvement of NTs in the brain–heart axis has recently started focusing especially on NTs. If the mechanism is fully revealed, an increase of NTs or neurotrophic factors can be a potential therapeutic strategy for improving both neuronal disorders and heart disease.

## Figures and Tables

**Table 1:** Distributional pattern and relative mRNA expression levels of NTs and their receptors in the mouse heart and vascular system obtained from RNA-seq results provided by the Deciphering the Mechanisms of Developmental Disorders (DMDD) Program. Yellow boxes and “+” indicate a higher mRNA expression; “±” lower expression; “−” no expression; blank denotes data not available.

□		E8	E9	E10	E11	E12	E13	E14	E15	E16	E17	E18	P0-3	P4-Adult
Heart	NGF	□	□	±	±	±	±	±	±	±	±	±	±	+
	BDNF	□	□	−	−	±	±	±	±	±	−	−	±	+
	NT-3	□	□	+	±	±	±	+	±	±	±	±	±	+
	NT-4/5	□	□	+	±	±	±	±	±	±	±	□	□	□
	TrkA	□	□	−	−	±	±	±	±	±	−	−	−	+
	TrkB	□	□	−	−	±	±	±	±	±	±	±	±	±
	TrkC	□	□	+	−	±	±	+	±	±	±	±	+	±
	p75NTR	□	□	+	±	±	±	+	±	±	±	±	±	+
Vascular System	NGF	□	□	□	□	±	□	□	□	□	□	□	□	±
	BDNF	□	□	□	□	□	□	±	□	□	□	□	□	±
	NT-3	□	□	□	□	□	□	±	□	□	□	□	□	±
	NT-4/5	□	□	□	□	□	□	□	□	□	□	□	□	□
	TrkA	□	□	□	□	□	□	□	□	□	□	□	□	±
	TrkB	□	□	±	□	□	□	□	□	□	□	□	□	±
	TrkC	□	□	±	+	□	□	±	±	□	□	□	□	±
	p75NTR	□	□	□	□	□	□	□	□	□	□	□	□	±

## Data Availability

All relevant data is presented within the manuscript.
